# The Performance of Healthcare-associated Infection Control Guideline among Hospital Nurses: A Structural Equation Model

**Published:** 2018-05

**Authors:** Jeong Eun MOON, Keum Seong JANG

**Affiliations:** 1. Chosun Nursing College, Gwangju, Republic of Korea; 2. College of Nursing, Chonnam National University, Chonnam Research Institute of Nursing Science, Gwangju, Republic of Korea

**Keywords:** Healthcare-associated infection, Nurse, Theory of planned behavior, Structural model

## Abstract

**Background:**

To improve efficient and systematic management and following of healthcare-associated infection guidelines, the relationship among various factors must be understood. Efforts should be made to strengthen or reduce relevant factors. We developed a structural equation model for following HAI-control guidelines

**Methods:**

Overall, 388 Korean registered nurses participated in this study and data collection was conducted Jul 21–Aug 31, 2014 using a self-reported questionnaire, and 392 of 400 copies were collected (98% recovery rate). Data were analyzed using descriptive statistics, reliability, and Pearson’s coefficient. Model identification evaluation was conducted by goodness-of-fit index and impact analysis.

**Results:**

Results of goodness-of-fit of modified model were as follows: χ^2^=120.57 (df=16, *P*<.001), GFI (Goodness of Fit Index)=.95, RMSEA (Root-Mean-Square Error of Approximation)=.10, SRMR (Standardizes Root-Mean-Square residual)=.06, NFI (Normal Fir Index)=.90, CFI (Comparative Fit Index)=.90. Factors with a statistically significant direct impact on behavioral intention are as follows; perceived behavior control (β=.35, *P*=.008), subjective norm (β=.27, *P*=.002), and job demands (β=−.08, *P*=.083). Organizational factors directly influenced following guidelines. Explanatory power of organizational factors on guideline compliance was greatest (C.R. [Critical Ratio] =5.67, *P*<.001).

**Conclusion:**

Supportive leadership and a positive organizational culture development strategy are essential. The hospital leader group should provide adequate resources to facilitate compliance with guidelines as well as create an atmosphere of on-site support that ensures guidelines will be followed, and engage in continuous monitoring and feedback regarding following of guidelines.

## Introduction

Prevention and control of healthcare-associated infections (HAIs) are very important topics in the recent healthcare environment. Despite the efforts of many organizations to reduce HAIs, HAIs are still increasing due to the increase in elderly population, chronic degenerative diseases, and immunocompromised patients as well as treatment with anticancer drugs and immunosuppressive therapies, the increase in antibiotic-resistant microorganisms, and advances in invasive procedure (1, 2).

Nevertheless, HAIs management has been limited to studies on the relationship between information awareness of certain microorganismal guidelines and the actual proliferation of those specific microorganisms (3–5); studies of the factors affecting guidance in operating rooms or intensive care units (6,7); and studies of the hand-hygiene practice among the various guidelines (8–13).

The provision of sufficient resources at the organizational level (8), the beliefs of the nurse concerning behavior (12), and the strength of work at the job site (13) as influencing factors in infection-control activities. Intensive education for the improvement of individual implementation of the guidelines, the necessity of observation and feedback, and evidence-based knowledge are needed along with a positive attitude and social awareness towards the behavior (8,11). In other words, in order to improve the efficient and systematic management and practice of HAIs guidelines, it is necessary to grasp the relationship among various factors at an individual, job, and organizational level. Moreover, efforts should be made to either strengthen or reduce the relevant factors.

Here in, it is necessary to identify the relationship between the relevant factors at the individual, job, and organizational levels in order to facilitate the understanding of the performance of HAI control guidelines by hospital nurses. In addition, considering the recent domestic healthcare situation that is increasingly interested in improving HAIs management guidelines, a causal study of the factors influencing the practice of HAIs management guidelines may be useful in establishing and applying effective strategies to enhance the practice of future guidelines.

We constructed a conceptual framework based on Ajzen’s *Theory of planned behavior* (TpB) (14) and on the literature review. TpB is a proposed theory (15) that can explain and predict various human social behaviors. It describes the relationship between attitudes, behavioral intentions, and behaviors, in order to understand human behavior in various disciplines including nursing. Our conceptual framework was constructed based on individual attitudes and perceived behavioral control (PBC) are defined as individual characteristics. Subjective norms are defined as a job characteristics’ dimension because social actors influence individuals in closed societies such as teachers, doctors, parents, and brothers by means of these norms (14). However, in TpB, social factors are factors in the narrow sense, which means they represent the group of field to which the individual belongs. Therefore, various societal approaches are needed for actions completely controlled by the will of the individual (15).

The framework, including objective knowledge, job demands, resource supports, and organizational culture, were used to construct the TpB.

The purpose of this study was to provide a foundation for individual, job, and organizational strategies to promote the performance of HAI control guidelines for hospital nurses by developing a structural model.

## Materials and Methods

### Participants and data collection

Sixteen general or tertiary hospitals were obtained by convenience sampling. According to the cooperation of the institution, 20–30 nurses were obtained by convenience sampling in order to include medical wards, surgical wards, Intensive Care Unit (ICUs), and emergency departments. Considering the drop-out rate, 350–400 subjects were selected based on 200–400 recommendations for multivariate statistical analysis, which involved use of the IBM SPSS Amos 21.00 (IBM Corp., Build 1178) (16).

Data collection was conducted from Jul 21, 2014, to Aug 31, 2014, using self-report questionnaire through direct visits and mailing surveys, and 392 copies out of the distributed 400 copies were collected (98% recovery rate). The 392 copies were checked for missing items and outliers to confirm the accuracy of the data entry and 4 data sets with more than 10% missing were rejected (16). The final 388 datasets were used for analysis.

### Study tools

We modified the selected study tools with the consent of the developers. We tried to confirm the validity and reliability of the study tools by repeating the preliminary survey twice for the 25 nurses of the university hospitals in accordance with the content-validity test of the expert group and the target selection criteria. The expert group consisted of 6 experts; three professors of the nursing department, one professor of the division of infectious diseases, and two infection-control practitioners worked for at least three years in an infection-control department in the hospital. Content validity tests and preliminary surveys were conducted to remove and modify the items with a *Content Validity Index* (CVI) of less than 80% and a Cronbach’s α of less than .70 (16). Therefore, the final 161 items were identified.

### Concepts of TpB for the performance of the HAI control guidelines

The concepts were written according to the questionnaire composition strategy using TpB (17). In order to measure the attitude (Cronbach’s α .70; 5 items), PBC (Cronbach’s α .81; 5 items), and subjective norm (Cronbach’s α .76; 2 items) for performance and to measure the intention (Cronbach’s α .80; 3 items) to perform guidelines, we modified the Jeong & Kim’s attitude instrument for hand-hygiene behavior (each other Cronbach’s α; .75, .84, .87, .86) (9). A Likert scale was used for the rest of the items, except for the attitude tools, which used a 7-point semantic differential scale.

### Objective knowledge

In order to measure the degree of objective knowledge on HAI management methods and procedures, we modified the cognitive measurement tools for BSI and UTI management guidelines (18), a measurement tool for hand-hygiene knowledge (2), and guidelines for preventing healthcare-associated pneumonia (19); we composed 60 items. One point was awarded for each correct answer, and in the case of a wrong answer or no answer, the score for that item was converted into 0 points.

### Job demands

In order to measure the time pressure, workload increase, work stoppage, and degree of job burden for performance of guidelines, 3 items were composed with a Likert 7-point scale (Cronbach’s α =.76), and we modified the Korean job stress measurement tool shortening (Cronbach’s α= .71) (20).

### Organizational factors

In order to measure organizational values, beliefs, and behaviors affecting the thinking and behavior of organizational members for carrying out guidelines, we modified Park’s (21) patient safety culture measurement tool (Cronbach’s α= .83). The reliability of the organizational culture measurement tool was .85. And in order to measure the degree of administrative and support, such as the training of employees and the support of related goods provided by the institution for the establishment, application of policies on infection control, we developed five items (Cronbach’s α = .81).

### Performance

To assess the degree of performance of guidelines, we composed 49 items based on the guidelines of Center for Disease Control and Prevention (CDC) (19), World Health Organization (WHO) (22), and Kim’s evaluation tool (18). All five items about PBC and 4th item about organizational-culture were presented in reverse conversion because they were negative type items.

### Ethical aspects

This study was endorsed by the CNUHH Institutional Review Board (IRB No. CNUHH-2014-020). The questionnaire was completed only by those who voluntarily agreed to participate in the study.

### Analysis method

The collected data were analyzed using IBM SPSS Statistics 21.0 (IBM Corp., Armonk, NY) and IBM SPSS Amos ver.21.00 (IBM Corp., Build 1178) programs.

The normality was verified by analyzing the skewness and kurtosis. The general characteristics of the participants were analyzed using descriptive statistics. The correlation between the measured variables was analyzed by Pearson’s coefficient. In order to confirm the estimation possibility of the *structural equation model* (SEM), the model identification evaluation was performed. The goodness-of-fit of the model was confirmed using the χ^2^ statistic and GFI (Goodness of Fit Index), SRMR (Standardizes Root Mean-square Residual), RMSEA (Root-Mean-Square Error of Approximation), NFI (Normal Fit Index), and CFI (Comparative Fit Index) (16). Statistically significant criteria are as follows: χ^2^ (*p*≥05), GFI ≥.90, SRMR ≤.08, RMSEA ≤.05, NFI ≥.90, CFI ≥.90. Parameter estimation and effect analysis were performed using the bootstrapping method. Statistical significance was set at *P*<0.05.

## Results

### The test of normality, correlation, and validity

Table 1 shows the general characteristics of participants. We conducted a descriptive statistical analysis to confirm the normality of collected data before the hypothesis test (Table 2). Univariate normality was satisfied (16). However, the multivariate kurtosis index was not satisfied at a significance level of .05, so we used the bootstrapping method. The absolute value of the correlation coefficient between measured values is in the range of .05∼.67, therefore, there was no problem of multicollinearity (16).

**Table 1:** General Characteristics of Participants

***Characteristics***	***Categories***	***n***	***%***	***M (SD)***
Age(yr)	<30	206	53.1	31.35 (7.61)
	30∼39	120	30.9	
	40∼49	47	12.1	
	≥50	15	3.9	
Career (year)	<1	18	4.6	8.36 (7.60)
	1∼ < 5 yr	149	38.4	
	5∼ <10 yr	93	24.0	
	10∼ < 15yearls	55	14.2	
	≥15	73	18.8	
Department in hospital	Medical ward	66	17.0	
	Surgical ward	105	27.1	
	ICU	86	22.2	
	ER	51	13.1	
	Cancer ward	62	16.0	
	Others^a^	18	4.6	
Hospital scale	General hospital	107	27.6	
	Tertiary hospital	281	72.4	
No. of beds	500∼ 999	332	85.6	913.57(521.31)
	1,000 ∼ 1,499	19	4.9	
	≥ 1,500	37	9.5	

a Others; psychiatric ward, isolation ward and transplantation ward // (*N=388*)

**Table 2:** Descriptive Statistics of Observed Variables

	***M(SD)***	***Min***	***Max***	***Skewness***	***Kurtosis***
Objective knowledge	0.76 (0.06)	0.51	0.91	−0.31	0.39
Attitude	6.79 (0.39)	5.00	7.00	−2.07	3.91
Perceived behavior control	4.97 (1.08)	2.00	7.00	−0.38	−0.42
Subjective Norm	6.44 (0.53)	5.00	7.00	−0.48	−0.62
Job demands	4.19 (1.31)	1.00	7.00	−0.24	−0.44
Intention	5.77 (0.80)	3.33	7.00	−0.48	−0.13
Organizational culture	5.51 (0.75)	3.30	7.00	−0.29	−0.38
Resource supply	5.91 (0.78)	3.60	7.00	−0.68	−0.09
Behavior [Performance]	4.55 (0.43)	1.00	5.00	−2.48	9.98

(*N*=388)

The *Confirmative Factor Analysis* (CFA) was performed to examine the reliability and validity of the measurement model. The CFA result for the initial measurement model showed that the goodness-of-fit of the model was χ^2^=28.30(df=7, *P*<.01), GFI (Goodness of Fit Index)=.96, RMSEA (Root-Mean-Square Error of Approximation)=.09, SRMR (Standardizes Root Mean-square Residual)=.02, NFI (Normal Fit Index)=.97, and CFI (Comparative Fit Index)=.98, which met the recommended standard (16). As a result of the measurement model test, factor loadings were .60∼.92, AVEs (Average Variance Extracted) were .45–.79, and construct reliability was .77–.89, which met the criteria for convergent validity of more than .70. In addition, *squared multiple regression* (SMR)[R^2^] between the perceived organizational-culture and resource support, which showed the greatest correlation, was .44. This value did not exceed the minimum AVE .45, confirming that the discriminant validity between each latent variable was satisfied (16).

### Test of hypothetical model, model modification

**Fig. 1:**
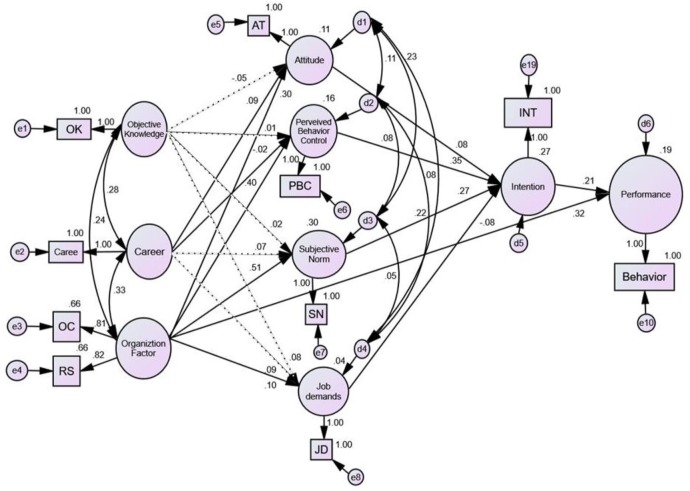
Path diagram for the modified model OK=Objective Knowledge; Caree=Career; AT=Attitude; PBC=Perceived Behavioral Control; SN=Subjective Norm; JD=Job Demands, OC=Perceived Organizational-Culture; RS=Resource Supply; INT=Intention

In order to confirm the validity of the proposed causal model, the goodness-of-fit of the hypothetical model was tested using the covariance structure analysis. Only GFI met the criteria (χ^2^=144.77 (df=17, *P*<.001), GFI=.94, RMSEA=.14, SRMR=.09, NFI=.84, CFI=.85). Therefore, it was necessary to try to modify the model by *modification indices* of the AMOS program and the theoretical background. We modified the model by adding the path between ‘Organizational factors→Performance’ according to the model modification method of the AMOS program (Fig. 1) (16).

The test results of goodness-of-fit of modified model were as follows (Table 3): χ^2^=120.57 (df=16, *P*<.001), GFI=.95, RMSEA=.10, SRMR=.06, NFI=.90, CFI=.90. The other goodness-of-fit indices were satisfactory except for the χ^2^ statistic, known to be sensitive to sample size and not recommended. The modified model of this study lost one degree of freedom with the addition of the path between ‘Organizational factors→Performance’. However, this is a nested model for a hypothetical model in which the χ^2^ value decreases statistically (*P*<.001) from 144.77 to 120.57. In addition, the SMC for ‘Performance’ increased from .14 to .19 according to the path addition, so we adopted the modified model as the final model.

**Table 3:** Goodness-of-fit of the Modified Model

***Model***	***χ^2^(*p*)***	***(df)***	***GFI***	***RMSEA***	***SRMR***	***NFI***	***CFI^a^***
Criteron	Low (≥.05)	≥.00	≥.90	≤.05	≤.08	≥.90	≥.90
Hypothetical model	144.77 (<.001)	17	.94	.14	.09	.84	.85
Modified model	120.57 (<.001)	16	.95	.10	.06	.90	.90

(*N*=388) //

a GFI=goodness of fit index; RMSEA=Root mean square error of approximation; SRMSR=Standardized root mean square residual; NFI=Normed fit index; CFI=Comparative fit index

Table 4 shows the effect analysis of the modified model. The direct effect of the nurses’ career (β=.09, *P*=.046) and organizational factors (β=.30, *P*=.004) on attitudes towards performance, the direct effect of organizational-factors (β=.40, *P*=.007) on the PBC, the direct effect of organizational factors (β=.51, *P*=.004) on the subjective norm, and the direct effect of organizational-factors (β=.10, *P*=.119) on job demands were statistically significant.

**Table 4:** Effects of the Modified Model

***Variables***	***Direct effect***	***Indirect effect***	***Total effect***	***SMC^a^***
	β (*p*)	β (*p*)	β (*p*)	
Attitude				.11
Objective knowledge	−.05 (.317)	-	−.05 (.317)	
Career	.09 (.046)	-	.09 (.046)	
Organizational factor	.30 (.004)	-	.30 (.004)	
Perceived behavioral control				.16
Objective knowledge	.01 (.944)	-	.01 (.944)	
Career	−.02 (.694)	-	−.02 (.694)	
Organizational factor	.40 (.007)	-	.40 (.007)	
Subjective norm				.30
Objective knowledge	.02 (.109)	-	.02 (.654)	
Career	.07 (.149)	-	.07 (.149)	
Organizational factor	.51 (.004)	-	.51 (.004)	
Job demands				.04
Objective knowledge	.08 (.654)	-	.08 (.109)	
Career	.09 (.123)	-	.09 (.123)	
Organizational factor	.10 (.119)	-	.10 (.119)	
Intention				.27
Attitude	.08 (.105)	-	.08 (.105)	
Perceived behavioral control	.35 (.008)	-	.35 (.008)	
Subjective norm	.27 (.002)	-	.27 (.002)	
Job demands	−.08 (.083)	-	−.08 (.083)	
Behavior				.19
Organizational factor	.32 (.005)	.06 (.008)	.38 (.007)	
Intention	.21 (.007)	-	.21 (.007)	

(*N*=388) //

a SMC, Squared multiple correlations

For the intention toward behavior, the direct effect of the factors such as the attitude (β=.08, *P*=.105), PBC (β=.35, *P*=.008), subjective norm (β=.27, *P*=.002), and job demands (β=−.08, *P*=.083) was statistically significant, and the explanatory power of these variables was 27%. For the performance of the HAI control guidelines, the direct effect (β=.32, *P*=.005), indirect effect (β=.06, *P*=.008), and total effect (β=.38, *P*=.007) of the organizational factor, and the direct effect of the intention were statistically significant (β=.21, *P*=.007). The explanatory power of these variables on the practice of the HAI control guidelines was 19.0%.

## Discussion

We discussed the goodness-of-fit of the model and influence factors on the performance of HAI control guidelines.

First, in the goodness-of-fit test of the final modified model, the absolute fit index (GFI, RMSEA, and SRMR) and the incremental fit index (NFI and CFI) met the criteria, indicating that the model-fit well with the data (16). The modified model is appropriate as a model for the performance of HAI control guidelines in hospital nurses. In addition, 27.0% of intention is explained by attitude, PBC, subjective norms, and job demands; and 19.0% of performance is explained by organizational factors and intentions. These results are somewhat lower than those reported (15), in which the explanatory power of TpB toward self-reported behavior was 31%. However, our results are higher compared with the results of the study on hand-hygiene practice (11%∼15%). Therefore, the modified model could be considered to be a suitable model to explain and predict the performance of HAI control guidelines.

Second, the organizational-factors had a stronger predictive power than does the intention toward performance of the guidelines (C.R=5.67, *P*<.001). Organizational factors had a positive effect on the intention through attitude, PBC, and subjective norm toward the performance of guidelines. In the modified model, organizational factors had a direct significant effect on the performance. Because there is no nursing literature describing TpB by inputting organizational factors, a direct comparison with this study was not possible. However, the context is as similar to codes of ethics (C.R=0.22, *P*<.01), ethical standards (C.R=0.36, *P*<.001), and organizational loyalty (C.R=0.29, *P*<.001) that constitute organizational-culture positively influence attitudes and intentions (23). We found that the propensity or character of an individual is influenced by the culture that the organization pursues. A few studies corroborate this result; strengthening leadership and commitment to helping employees in infection-control programs led to adherence to the guidelines(95% confidence interval, 3.53–4.23; *P*<.001) (8). Providing organizational resources and aggressive programs have improved hand hygiene practices from 66% to 97% (10). The comparatively stronger behavioral atmosphere of the department and the influence of senior physicians (β=3.00) and nursing managers (β=2.20) were more significant than the influence of individual attitudes(β=0.84) on the practice of hand- hygiene (12). Nurses who act as members of an organization can change their behaviors according to organizational values, goals, and behavioral directions. Therefore, it is important to strengthen the positive attitude of the individual and the desirable subjective norm for compliance with the HAI control guidelines. However, from a more efficient and long-term viewpoint, a supportive leadership and a positive organizational culture development strategy are comparatively more important.

Third, the explanatory power of intention was 27% and that of performance of guidelines was 19%. In a previous study that used TpB alone to describe infection-control activities (7,13, 24), the explanatory power of the intention varied from 35% to 56% for hand-hygiene and was about 45% in the study on wearing gloves for HIV prevention. The explanatory power of behavior is reported as 15% in hand hygiene studies and 61% in the study on wearing gloves. The performance of self-protection is much higher than other infection control practices, and the actual performance is low even when the intention is high. However, because there are few studies on the application of TpB in the field of HAI control, and because the explanatory power of intention and behavior varies in related studies, it is necessary to improve the explanatory power of the model by further considering the variables besides TpB constitution concepts; such as nursing competence, job satisfaction, and empowerment.

Finally, the performance of guidelines is a strong social characteristic. Stronger social characteristics have a stronger influence by the subjective norm or the PBC than the attitude (14), so we constructed an SEM in an extended form of TpB by introducing new variables. Such variables include social and organizational factors (10,13); career (5) and knowledge (6) that can affect an individual’s practice. However, in this study, the objective knowledge about the guidelines did not affect the TpB constructs, which showed different results from the previous studies that suggested positive correlation between the degree of knowledge and infection-control practice and attitude (3,6). However, the above studies cannot be directly compared to the results of this study for several reasons: They are limited to general hospitals or ICU; the assessments of knowledge increases were measured within one month after training; and the subjective knowledge that participants thought they knew to some extent, rather than the objective knowledge to judge the actual answer was measured. The objective knowledge measurement tool used in this study is a tool that develops the details presented in the latest evidence-based international guidelines. However, how the latest guidelines are reflected in the guidelines and policy of each hospital is unclear; and the degree of education and the type of education provided may vary by hospitals. In addition, the concepts of TpB measured the perception level of individuals, and the subject’s responses predominately favored infection-control practices are social behaviors. The relationship between the level of objective knowledge and the concepts perceived by the individual is not significant, similar to another study (13). Therefore, it is necessary to introduce a program that is a mixture of education and periodic practice feedback and conduct a comparative study through application of educational method that can raise actual knowledge based on behavioral theory. It is necessary to perform repeated studies to measure and compare objective and subjective knowledge together in the future.

## Conclusion

This study is the first model construction study conducted to explain the comprehensive HAI control guidelines behavior of hospital nurses. The predictive variables included in the model are personal attributes (objective knowledge, career, attitude, PBC), job attributes (subjective norms and job demands), and organizational attributes (organizational-culture and resource support). The predictive nature of the job and organizational attributes are limited compared to the personal attributes, it is necessary to improve the explanatory power of the model by further considering the variables such as nursing competence, job satisfaction, and empowerment.

## Ethical considerations

Ethical issues (Including plagiarism, informed consent, misconduct, data fabrication and/or falsification, double publication and/or submission, redundancy, etc.) have been completely observed by the authors.
